# Serum Pro-Inflammatory Cytokines and Leptin as Potential Biomarkers for Treatment Response and Toxicity in Locally Advanced Squamous Cell Carcinoma of the Head and Neck

**DOI:** 10.3390/diseases12030055

**Published:** 2024-03-11

**Authors:** Amani A. Alrehaili, Amal F. Gharib, Maha M. Bakhuraysah, Afaf Alharthi, Ohud Alsalmi, Fouzeyyah Ali Alsaeedi, Reem Ali Alhakami, Kamilah Ali Alasmari, Nuha Mohammed, Wael H. Elsawy

**Affiliations:** 1 Department of Clinical Laboratory Sciences, College of Applied Medical Sciences, Taif University, P.O. Box 11099, Taif 21944, Saudi Arabia; amgharib@tu.edu.sa (A.F.G.);; 2 Laboratory and Blood Bank, King Faisal Medical Complex, Ministry of Health, Taif 26524, Saudi Arabia; 3 Department of Clinical Oncology, Faculty of Medicine, Zagazig University, Zagazig 7120001, Egypt

**Keywords:** head and neck squamous cell carcinoma, cytokines, treatment response, biomarkers

## Abstract

Squamous cell carcinoma of the head and neck (HNSCC) is a globally prevalent form of cancer with significant morbidity and mortality rates. The present study examines the relationship of serum pro-inflammatory cytokines and leptin levels with the effectiveness of therapy in individuals with HNSCC and their potential role as biomarkers for treatment response and toxicity. Induction chemotherapy and concomitant chemoradiotherapy were evaluated for efficacy and safety in 52 individuals with HNSCC. Both response and toxicity were evaluated, and serum levels of pro-inflammatory cytokines Interlukin-1 beta (IL-1β), Interlukin-2 (IL-2), Interlukin-6 (IL-6), and Tumor Necrosis Factor-Alpha (TNF-α) and leptin were measured using enzyme-linked immunoassay before and after treatment. Before treatment, these measurements were made in comparison with a control group with 50 healthy people. The results showed that serum cytokines and leptin levels varied depending on the response to treatment, with patients who had a complete or partial response (PR) showing significant decreases in IL-1 β, IL-6, and TNF-α levels and significant increases in IL-2 and leptin levels after treatment, with an improvement in cachexia. These results imply that variations in serum pro-inflammatory cytokines and leptin levels are likely related to the therapeutic effectiveness in HNSCC and may act as biomarkers for treatment response.

## 1. Introduction

Concurrent chemotherapy plus radiotherapy improves results in head and neck squamous cell carcinoma (HNSCC) compared to radiation therapy alone [1], according to multiple randomized trials and recent meta-analyses [2,3]. Concurrent chemoradiotherapy is an established therapeutic option for advanced HNSCC [4]. In randomized trials, concurrent chemo- and radiotherapy improved survival rates [5,6]. These promising results suggest that this combination can enhance local and regional disease control in the head and neck, the most common region of relapse [7]. Traditionally, the primary goal of advanced HNSCC treatment was to preserve the patient’s organs and function [8]. Administering early chemotherapy is crucial in reducing cancer size, enabling organ-preserving surgery, and eliminating micro-metastases, according to Cohen et al. [6].

The cytokine interleukin 1 beta (IL-1β) plays a vital role in promoting inflammation and immune responses. In cases of HNSCC, IL-1β stimulates tumor growth and invasion and activates immune cells. It is an essential factor in understanding the development and progression of HNSCC [9]; it plays a crucial role in inflammation, has been observed to negatively affect the immune system’s ability to combat tumors in the HNSCC microenvironment, and plays a significant role in initiating and amplifying immune responses by inducing the expression of other pro-inflammatory cytokines, chemokines, and adhesion molecules. IL-1β can disrupt the function of immune cells such as cytotoxic T cells and natural killer (NK) cells [10]. Researchers have investigated IL-1β signaling as a therapeutic approach for HNSCC treatment [11].

Interleukin 2 (IL-2), an immunostimulatory substance, plays a pivotal role in enhancing the immune system by facilitating the proliferation and survival of T cells. This multifaceted cytokine not only promotes the growth of activated CD4+ and CD8+ effector T cells but also amplifies the cytotoxicity of NK cells [12]. Additionally, IL-2 exhibits the capacity to elevate the proliferation of B cells and enhance their antibody secretion. Overall, IL-2 serves as a crucial regulator by orchestrating various immune responses to combat antigens and maintain immune homeostasis [13].

Interleukin 6 (IL-6), a pro-inflammatory cytokine, plays an important role in inflammation, immune regulation, and cell proliferation. In HNSCC, IL-6 has been implicated in tumor development and progression. It substantially influences the progression of HNSCC through diverse mechanisms [14]. First, IL-6 facilitates tumor cell proliferation and survival by activating signaling pathways that promote cell growth and impede apoptosis [11]. Second, it stimulates angiogenesis and thus fosters the formation of new blood vessels to support tumor growth. Third, IL-6 impacts the anti-tumor immune response in HNSCC [15]; it regulates the recruitment of immune cells like myeloid-derived suppressor cells and regulatory T cells, which suppress effector immune cells, such as cytotoxic T cells and NK cells, leading to immune evasion by the tumor [16].

Tumor necrosis factor (TNF-α) is a cytokine that promotes inflammation and immune responses. In cases of HNSCC, it plays a key role in tumor development, progression, and immune modulation [17]. High levels of TNF-α have been observed in both the tumor tissue and the circulating blood of cancer patients [18]. It stimulates cancer cell proliferation, angiogenesis, tissue remodeling, and invasion, thus promoting tumor growth and progression [19]. Furthermore, TNF-α influences immune responses by regulating immune cell recruitment and activation. It can create an immunosuppressive environment by promoting the generation of regulatory T cells and myeloid-derived suppressor cells, contributing to tumor immune evasion [20].

Leptin, a hormone produced by fat cells, has been studied in relation to cancer; it regulates processes like appetite, metabolism, and energy balance [21]. Leptin also affects immune function, blood vessel formation, and cell growth. It can stimulate cancer cell growth and create an environment that supports tumor survival [22].

Obesity, a known risk factor for cancer, can indirectly affect cancer progression through its association with leptin. In obese individuals, higher levels of leptin are produced by fat cells, contributing to pro-cancer effects [23]. Leptin can also interact with other pathways, such as insulin-like growth factor 1 and estrogen, that further promote cancer growth [24].

Understanding the relationship between cytokine levels and therapy outcomes could have significant clinical implications. It may help identify individuals who are more likely to respond favorably to specific treatments, allowing for personalized therapeutic approaches. Additionally, it could contribute to the development of novel therapies targeting cytokines to improve treatment efficacy and reduce adverse effects in individuals with HNSCC.

The present study evaluates the outcomes of patients with HNSCC who received induction chemotherapy before undergoing concurrent chemotherapy, radiotherapy, and conservative surgery. The study investigates the relationship between cytokine levels (IL-1β, IL-2, IL-6, TNF-α, and leptin) as predictors of treatment response in HNSCC patients.

## 2. Patients and Methods

Fifty-two patients diagnosed with locally advanced, non-metastatic HNSCC admitted to the Clinical Oncology Department of Zagazig University in August 2018 and were subsequently enrolled. The study received approval from the Ethics Committee of Zagazig University (2018-Feb-214). Before participating in the study, all patients were required to sign a written informed consent form. To be eligible, patients had to have a histologically confirmed diagnosis of HNSCC without any evidence of metastasis, per the American Joint Committee on Cancer Staging criteria [25]. Patients were also required to be under 65 years old, have an Eastern Cooperative Oncology Group (ECOG) performance status of 0 or 1 [26], and possess normal cardiac, hepatorenal, and bone marrow functions. Blood samples were collected at the beginning of the study and during the assessment of the treatment response. The primary assessment involved reviewing the patient’s medical history, conducting a physical examination, and performing standard laboratory tests such as a complete blood cell count and routine serum chemistries. Additional diagnostic tests may have been performed, such as a chest X-ray, computerized tomography scan, or head and neck magnetic resonance imaging (MRI). Additionally, a triple endoscopy was undertaken to determine the size of the primary lesion and how far it had spread locally. An age- and gender-matched control group of 50 healthy individuals was included for comparison of biochemical parameters before treatment.

### 2.1. Treatment Plan

#### 2.1.1. First Phase of Treatment: Induction Chemotherapy

Patients received induction chemotherapy, which consisted of administering 100 mg/m^2^ of Cisplatin on the first day of treatment, followed by a continuous infusion of 5-Fluorouracil at 1000 mg/m^2^ from day 1 to day 5. This regimen was repeated every 28 days for a total of three cycles. After finishing the third cycle, patients underwent a response evaluation during the second week using updated guidelines for assessing responses in solid tumors [27].

#### 2.1.2. Second Phase of Treatment: Radiotherapy and Concomitant Chemotherapy

After induction chemotherapy, patients were assessed for response and then treated with concurrent chemoradiotherapy. This treatment involved receiving 66 Gy of radiation over 6–7 weeks, combined with 100 mg/m^2^ of intravenous cisplatin on days 1, 22, and 43. Following concurrent chemoradiotherapy, patients were categorized based on their response. Those who achieved a complete response (CR) were not required to undergo any further treatment. However, patients who achieved the complete disappearance of the primary tumor but still had neck nodes underwent neck dissection. For those with a partial response (PR) at the primary tumor site, conservative surgery with organ preservation was performed as needed. Finally, patients who did not respond to the initial treatment or experienced disease progression were shifted to another type of treatment.

#### 2.1.3. Quality of Life Evaluation

The study utilized the European Organization for the Research and Treatment of Cancer Quality of Life Questionnaire (EORTC QLQ-C30) to evaluate the effect of therapy on head and neck cancer patients’ quality of life [28]. Before and after treatment, a questionnaire was provided to patients to evaluate the therapy’s effectiveness in addressing symptoms. Each symptom was scored individually based on the change in its relative score. If there was an increase of one point or more in the score of a complaint during treatment, it was classified as “worse”. If it increased by two or more points, it was categorized as “much worse”. If the symptom decreased by one point or more, it was classified as “improved”. If it fell by two or more points, it was branded as “much improved”. This method quantifies changes in symptoms and provides valuable insights into a treatment’s efficacy in addressing specific symptoms.

#### 2.1.4. Evaluation of the Levels of Serum Pro-Inflammatory Cytokines and Leptin

Pro-inflammatory cytokines (IL-1ß, IL-2, IL-6, and TNF-α) and leptin were measured in serum before and after treatment and compared with the control group before treatment. The pro-inflammatory cytokines were detected using a sandwich enzyme-linked immunoassay (ELISA) test that utilized monoclonal antibodies targeting two different epitopes on the cytokine molecule. The IL-1β and IL-2 human ELISA kits (0.05 pg/mL sensitivity for IL-1β and 9.1 pg/mL for IL-2) were manufactured by Thermo Fisher Scientific (Invitrogen) (Frederick, MD, USA), with catalog numbers KHC0011 for IL-1β and BMS221-2 for IL-2. Meanwhile, the ELISA kits for IL-6 (sensitivity: 1.6 pg/mL) and TNF-α (sensitivity: 1.6 pg/mL) detection were obtained from Abcam (Cambridge, UK), with catalog numbers ab178013 for IL-6 and ab285312 for TNF-α. Serum leptin levels were measured using the human leptin ELISA kit manufactured by Abcam (catalog number: ab108879).

### 2.2. Statistical Analysis

The SPSS software package (v. 27; IBM, Chicago, IL, USA) was employed for data analysis, with Student’s *t*-tests used to compare means and standard deviations between different groups. The Kaplan–Meier curve was employed to estimate the survival rate in HNSCC patients, and the log-rank test was used to evaluate the significance of survival differences.

## 3. Results

The study population comprised 52 patients diagnosed with HNSCC, as illustrated in Table 1, which provides information on patient gender, age, and tumor characteristics, such as location, grade, and clinical stage. Additionally, the table includes details on patients’ ECOG performance status—that is, their ability to perform daily activities—and notes their degree of weight loss at the beginning of treatment. The study comprised 39 male (75%) and 13 (25%) female patients, with a mean age of 54.2 ± 16.24 (median: 50.4 years). There was a statistically significant difference between leptin levels for males and females in LSCC and controls, as determined by analysis of variance (ANOVA) analysis (*p* = 0.0362). Cancer was most commonly found in the oropharynx (42.3%), followed by the hypopharynx (30.7%), larynx (19.3%), and the oral cavity (7.7%). Moderately differentiated tumors (42.3%) were the most common, followed by poorly differentiated (34.6%) and well-differentiated (23.1%) tumors. Most patients (92.4%) had stage IV disease, with T4N2 being the most common, and four-fifth of patients (80.8%) had an ECOG performance status of 0, indicating good overall health. Weight loss was observed in some patients: 57.7% showed no weight loss, and 23% had weight loss greater than 5% from the start of treatment.

### 3.1. Response to Treatment

All 52 patients received induction chemotherapy, but two were not evaluated for response: one patient (1.9%) died early, and another (1.9%) was shifted to alternative therapy due to therapy-related toxicity during induction chemotherapy (Table 2).

After three cycles of induction chemotherapy, clinical responses for the remaining 50 patients were evaluated. The results showed that only 16% of patients had a CR (i.e., disappearance of the tumor), while 36% had a PR; the tumor was reduced in size but did not disappear completely. Another 36% of patients had stable disease (SD), indicating no significant change in tumor size, and 12% had progressive disease (PD), which means that the tumor increased in size. After receiving chemotherapy and chemoradiotherapy, 44 patients were evaluated for response. Among them, 59.1% achieved a CR, 15.9% had a PR, 13.6% had SD, and 11.4% had PD.

After three cycles of induction chemotherapy, concomitant chemoradiotherapy, and surgery, the clinical response data for the evaluated patients were as follows: 59.1% of patients achieved a CR, and two of seven initially had a PR but refused conservative surgery as part of treatment. SD was found in two of five patients who were not suitable candidates for radical surgery for medical reasons and in two other patients who passed away after concomitant chemoradiotherapy. Three patients with PD shifted to other treatments.

After treatment, 59.1% of patients achieved a CR, meaning that their head and neck organs were preserved and that no disfiguring radical surgeries were required. While some patients had a PR and refused surgery, others were unsuitable for radical surgery for specific reasons and had SD; two patients passed away after treatment. A small number of patients were shifted to other forms of treatment.

### 3.2. Body Mass Index before and after Treatment

Evaluation of BMI in controls and LSCC patients before treatment and in those with responses to treatment revealed a statistically significant relationship among the three groups (F factor: 26.07, *p* < 0.0001).

### 3.3. Treatment Toxicity

Table 3 displays the toxicity grades of patients who underwent induction chemotherapy for HNSCC. The toxicity grades range from 0 (no toxicity) to IV (severe toxicity).

For hemoglobin levels, the majority of patients (38.5%) experienced Grade I toxicity, followed by Grades II (19.2%) and III (19.2%), with no Grade IV cases.

Regarding granulocytes, Grade I toxicity was most prevalent (34.7%), followed by Grades II (19.2%) and III (15.4%). Grade IV toxicity was observed in 19.2% of patients. As for platelets, the highest percentage of patients experienced Grade II toxicity (26.8%), followed by Grades IV (19.2%) and III (15.4%). Most patients (76.9%) had Grade 0 toxicity regarding creatinine levels, indicating no adverse effects; Grades I, II, and III toxicities were observed in just a few patients (7.7% each), and none experienced Grade IV toxicity. Most (76.9%) experienced Grade 0 toxicity concerning nausea and vomiting, indicating no significant adverse effects. A small portion of patients (3.8%) experienced Grade I toxicity, followed by Grades II (11.6%) and III (7.7%); no patients experienced Grade IV toxicity. For diarrhea, the highest prevalence was Grade 0 (no diarrhea, 69.3%), followed by Grades III (15.4%), IV (7.7%), II (3.8%), and I (3.8%) toxicities. The toxicity grades of mucositis varied widely. The most prevalent toxicity grade was III (30.8%), followed by Grades I (15.4%), II (15.4%), IV (11.5%), and 0 (26.8%). Notably, no patients experienced neurotoxicity during induction chemotherapy for HNSCC.

Table 3 summarizes the toxicity grades of induction chemotherapy in HNSCC. The results indicate that the treatment was generally well tolerated in terms of creatinine, nausea and vomiting, and diarrhea. However, mucositis had a significant impact on a large proportion of patients. The absence of neurotoxicity is a positive finding.

Table 4 provides data on the toxicities experienced by patients during chemoradiotherapy.

### 3.4. Quality of Life Evaluation

Almost a third (31.6%) of patients reported less painful throat symptoms during treatment, while 47.4% of patients were less concerned about their appearance while receiving treatment. This could indicate that the therapy successfully improved a patient’s physical appearance or that a patient better understood the treatment process and its effects. One in five (21%) of patients experienced significant decreases in tooth-related difficulties during treatment; this could indicate that the therapy effectively managed cancer-related dental issues and their treatment. A small number (15.8%) of patients did not gain weight during treatment, which suggests that the therapy did not cause significant fluid retention or hormonal changes that might lead to weight gain.

### 3.5. Effects of Treatment on Serum Pro-Inflammatory Cytokines and Leptin Levels

Table 5 provides information on the serum levels of cytokines in healthy controls and patients with HNSCC. IL-1β levels were also elevated in patients with HNSCC, with a mean value of 12.4 ± 4.3 pg/mL, compared to 3.6 ± 1.2 pg/mL in healthy controls, indicating a highly statistically significant difference (*p* < 0.0001). Similarly, the levels of IL-2 were substantially higher in patients with HNSCC (14.9 ± 4.1 pg/mL) than in healthy controls (2.31 ± 1.1 pg/mL) (*p* < 0.0001). Patients with HNSCC also exhibited elevated levels of IL-6 (37 ± 14.9 pg/mL) compared to healthy controls (2.8 ± 1.5 pg/mL); the difference was highly statistically significant (*p* < 0.0001). TNF-α levels in patients with HNSCC (28 ± 12.9 pg/mL) were substantially higher than those in healthy controls (2.1 ± 1.7 pg/mL; *p* < 0.0001). Similarly, leptin levels in patients with HNSCC (19 ± 16.1 pg/mL) were considerably higher than those in healthy controls (3.1 ± 1.6 pg/mL; *p* < 0.0001).

The cytokine levels of patients with HNSCC before and after treatment varied with their response to treatment. Those who responded well to treatment (i.e., the CR + PR group) generally showed favorable changes in cytokine levels.

Table 6 presents data on cytokine levels (IL-1β, IL-2, IL-6, TNF-α) and leptin in patients with HNSCC before and after treatment, categorized by response to treatment.

**IL-1β:** Before treatment, the CR + PR group had an average IL-1β level of 44.3 ± 31 pg/mL, higher than the SD group (42.5 ± 2.1 pg/mL) and the PD group (28.7 ± 16.2 pg/mL). After treatment, the CR + PR group showed a significant decrease in IL-1β level to 17.3 ± 3.4 pg/mL, while the SD group had an average level of 25 ± 4.2 pg/mL, and the PD group had an average level of 34.3 ± 9.6 pg/mL.

**IL-2:** Before treatment, the CR + PR group had an average IL-2 level of 29.3 ± 11.7 pg/mL, while the SD and PD groups had average levels of 53.5 ± 40.3 pg/mL and 31.3 ± 19.1 pg/mL, respectively. After treatment, the CR + PR group significantly increased IL-2 levels to 61.7 ± 37.7 pg/mL. The SD group had an average level of 41.5 ± 4.9 pg/mL, and the PD group had an average level of 23.7 ± 5.5 pg/mL.

**IL-6:** Before treatment, the CR + PR group had an average IL-6 level of 32 ± 9 pg/mL, while the SD and PD groups had average levels of 51.5 ± 14.4 pg/mL and 19 ± 3.6 pg/mL, respectively. After treatment, the CR + PR group showed a decrease in IL-6 levels to 14.8 ± 5.7 pg/mL, but this difference was not statistically significant. The SD group had an average level of 32 ± 8.5 pg/mL, and the PD group had an average level of 22.5 ± 4.6 pg/mL.

**TNF-α:** Before treatment, the CR + PR group had an average TNF-α level of 43.7 ± 14.7 pg/mL, higher than the SD group (47.5 ± 10.6 pg/mL) and the PD group (28.3 ± 11.2 pg/mL). After treatment, the CR + PR group showed a significant decrease in TNF-α level to 23.2 ± 8.5 pg/mL. The SD group had an average level of 35.5 ± 7.8 pg/mL, and the PD group had an average level of 34.7 ± 9.1 pg/mL.

The cytokine levels in patients with HNSCC varied before and after treatment, depending on their response. The group with CR or PR generally exhibited changes in cytokine levels that indicated positive treatment response, such as lower levels of IL-1β and TNF-α.

**Leptin:** Before treatment, patients in the CR + PR group had an average leptin level of 10.7 ± 6.5 pg/mL, while those with SD and PD had average levels of 2.3 ± 2 pg/mL and 6.5 ± 4.2 pg/mL, respectively. After treatment, the CR + PR group showed an increased leptin level, averaging 16 ± 8.6 pg/mL. However, this increase was not statistically significant. The SD group had an average level of 5.6 ± 2.2 pg/mL, and the PD group had an average level of 6.1 ± 4.5 pg/mL.

### 3.6. Survival Outcomes in HNSCC Patients

**Figure 1:**
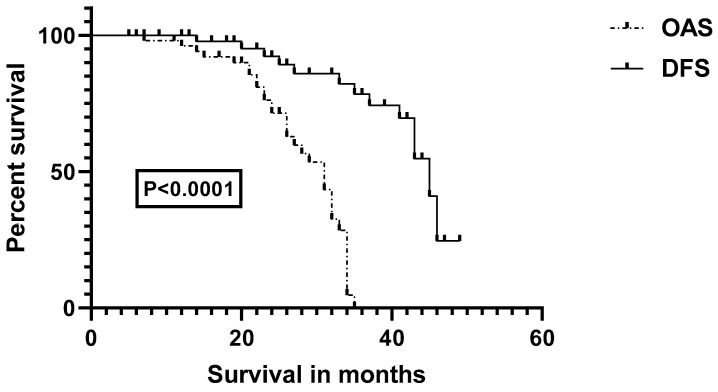
Impact of treatment response on survival in HNSCC patients. **OAS**, overall survival; **DFS**, disease-free survival.

The analysis of overall and disease-free survival outcomes in patients with HNSCC reveals significant differences. The log-rank (Mantel–Cox) and Gehan–Breslow–Wilcoxon tests both produced highly significant *p*-values (<0.0001), indicating variation in overall and disease-free survival rates among HNSCC patients. Median overall survival was 31 months, while disease-free survival was 45 months (Figure 1).

## 4. Discussion

There is no agreement on the optimal approach for treating or managing advanced HNSCC. Some medical professionals recommend a combination of surgery and radiation therapy, while others advocate radiation therapy alone. Ultimately, the optimal treatment for each patient will depend on individual factors, including the stage and location of the patient’s cancer, overall health, and personal preferences. Standard treatments for advanced head and neck cancer, such as radiation therapy, surgery, or a combination of the two, have a cure rate of less than 40%. However, recent research has shown that combining chemotherapy and radiation therapy (chemoradiotherapy) can improve the chances of survival for patients with advanced HNSCC [29]. Concurrent chemoradiotherapy is more effective than radiation therapy alone in improving survival rates for patients with HNSCC. It also allows for the preservation of organs but may increase the risk of acute and chronic toxicities. Some studies suggest that adding multiagent chemotherapy to concurrent chemoradiotherapy may further improve outcomes, although this may come with an increased risk of toxicity [30].

One of our aims is to increase local control and survival rates and allow organ preservation, a common goal in many studies investigating new treatment approaches for this disease. According to the British Columbia Cancer Agency, concomitant chemoradiotherapy is an effective treatment for HNSCC that can improve survival rates and reduce the risk of recurrence [31].

We found that a large majority of patients in our study (80.8%) had good performance status (ECOG 0), consistent with previous studies that have shown that patients with better performance status tend to have better treatment response and survival rates with HNSCC. For example, a multicenter retrospective study of patients with locally advanced HNSCC found that patients with ECOG scores of 0 or 1 had significantly better overall survival rates than those with ECOG scores of 2 or higher [32]. According to our results, most patients (57.7%) with HNSCC did not lose weight after beginning treatment. This is consistent with other studies that have found varying rates of weight loss among HNSCC patients. Studies analyzing weight loss in individuals with HNSCC reveal that the median weight loss at the start of treatment was 5.4%, consistent with the percentage of patients who experienced weight loss in our study. Reviews and meta-analyses support these findings. One study also found that weight loss was associated with poorer survival outcomes, highlighting the importance of addressing nutrition and weight management in the treatment of HNSCC [33].

The response rates after induction chemotherapy in our study are consistent with previous research on induction chemotherapy in HNSCC. In a randomized phase III trial, patients with HNSCC who underwent induction chemotherapy followed by concurrent chemoradiotherapy had an overall response rate of 59% [34]. Our study on the effectiveness of induction chemotherapy in HNSCC showed a CR rate of 16%, which aligns with the 16% CR rate found in a meta-analysis of relevant studies [35]. It is important to note that the definition of CR varies between studies, which may affect the reported rates.

The response rates observed in our study after induction chemotherapy and concomitant chemoradiotherapy are consistent with previous studies that have investigated this treatment approach in patients with HNSCC. Regarding CR rates, our study found a rate of 59% after induction chemotherapy and concurrent chemoradiotherapy, which is higher than the rates reported in some previous studies. It is worth noting that approximately 60% of our patients with CRs could avoid radical or mutilating surgeries and maintain normal organ function. A research study examined whether administering chemotherapy before and during radiation treatment was effective for advanced HNSCC patients. The study revealed that 41% of patients experienced a CR to the treatment [36]. However, it is important to note that the definition of CR may vary between studies, impacting the reported rates.

The outcome of patients in our study suggests that the treatment protocol used in the study may positively affect survival and progression-free survival in patients with head and neck cancer. Our findings are consistent with previous research on induction chemotherapy and concurrent chemoradiotherapy in patients with these forms of cancer.

A study conducted on people with advanced HNSCC found that induction chemotherapy followed by concurrent chemoradiotherapy resulted in a median overall survival time of 14 months and a median progression-free survival time of 8 months [37]. Other research concluded that individuals with locally advanced HNSCC who underwent induction chemotherapy and concurrent chemoradiotherapy had a median overall survival of 18 months and a median progression-free survival of 12 months [38].

The present study’s survival rates are higher than those reported in previous research, which may be due to differences in patient characteristics, treatment protocols, and follow-up periods. On the other hand, the response rate observed here is consistent with earlier studies, which suggests that induction chemotherapy and concurrent chemoradiotherapy may be effective treatment approaches for head and neck cancer patients. It should be noted that our results are limited due to a small sample size and a short follow-up period. Further research is needed to establish the long-term effectiveness and safety of induction chemotherapy and concurrent chemoradiotherapy in treating HNSCC.

The treatment protocol appears to be relatively well tolerated by patients, with most experiencing mild-to-moderate toxicity. These findings are consistent with previous studies investigating induction chemotherapy followed by concurrent chemoradiotherapy in patients with HNSCC [39]. A randomized phase III trial of induction chemotherapy followed by chemoradiotherapy in people with locally advanced HNSCC reported that the most common toxicities were nausea, vomiting, and mucositis, which affected more than half of patients [40]. Another study explored the effects of induction chemotherapy followed by chemoradiotherapy in people with advanced HNSCC; it found similar toxicity profiles, with hematologic toxicity, mucositis, and gastrointestinal toxicity as the most frequent side effects [41]. The lack of neurotoxicity observed in the present study is an important finding, as neurotoxicity can significantly impact patient quality of life and treatment outcomes. This suggests that the treatment protocol we used may be well tolerated by patients in terms of nervous system damage.

Overall, our study suggests the induction chemotherapy protocol may be a viable and well-tolerated treatment choice for HNSCC patients based on its observed toxicity profile. It is important to note that toxicity profiles can vary with factors like patient characteristics, type and stage of cancer, and the treatment protocols used. Therefore, additional research is required to establish the most effective approach for using induction chemotherapy and concurrent chemoradiotherapy to treat HNSCC. Additionally, future research should prioritize identifying the patients most likely to benefit from this treatment while minimizing the risk of harmful side effects.

The quality of life observed in our study is promising. The treatment protocol used may positively impact patients’ physical and emotional well-being. A study conducted to test the effectiveness of induction chemotherapy followed by chemoradiotherapy as a treatment for advanced head and neck cancer found that patients experienced significant improvements in quality of life, particularly in physical functioning, health status, and social functioning [40]. Another study examined how induction chemotherapy followed by chemoradiotherapy can benefit people with locally advanced HNSCC and showed that those who received this treatment reported an improvement in quality of life, particularly in the areas of physical and social functioning [37]. However, as noted above, the impact on quality of life may vary depending on factors like the type and stage of cancer, patient characteristics, and treatment protocols.

Our study’s results suggest that cytokine level changes may be linked to treatment response in patients with HNSCC; they show that, after treatment, patients with CR + PR had significantly decreased IL-1β, IL-6, and TNF-α levels and increased IL-2 and leptin levels. Patients before initial chemotherapy have significantly higher cytokine levels than healthy individuals (controls). The disease, tumor microenvironment, and chemotherapy treatment can all contribute to this. The elevated cytokine levels highlight the presence of immune dysregulation and an inflammatory response in patients with the underlying disease. These findings can help identify targets for therapeutic interventions to modulate the inflammatory response in these patients. Additionally, because of differences in patient populations, treatment protocols, and methods for measuring cytokine levels, the results of our study cannot be directly compared to those of other studies. Several studies have looked into the role of cytokines in the pathogenesis of HNSCC, investigating the changes in cytokine levels in patients with HNSCC in response to treatment and their association with treatment outcomes. Caruntu et al. investigated cytokine levels in patients with HNSCC before and after concurrent chemoradiotherapy and discovered that changes in cytokine levels were associated with treatment response and survival outcomes [42]. Reers and colleagues found that changes in cytokine levels significantly impacted the success of treatment and overall outcome for patients with HNSCC, both prior to and following intensity-modulated radiation therapy [43].

To summarize, the present study suggests that changes in cytokine levels (IL-1β, IL-2, IL-6, and TNF-α) could serve as biomarkers for predicting treatment response and prognosis in patients with HNSCC. Patients who responded positively to the treatment showed elevated leptin levels. Conversely, those with stable or worsening conditions encountered no notable leptin level fluctuations. Leptin is an adipokine that has been implicated in the development of cachexia, and several studies have investigated the relationship between leptin and cachexia in head and neck cancer patients. For example, Paval et al. found that cachectic patients had significantly lower leptin levels than non-cachectic patients [44]. Similarly, Muthanandam et al. found that decreased leptin levels were associated with a higher risk of cachexia in patients with advanced head and neck cancer [45].

The study’s results indicate that leptin may play a role in the development of cachexia in patients with HNSCC. The rise in leptin levels seen in patients who had a complete or partial response to treatment suggests a possible improvement in their nutritional status and a decrease in cachexia. We observed a significant difference in leptin levels between male and female patients with LSCC (*p* = 0.0362). This difference can be attributed to variations in body fat distribution and hormonal regulation of appetite and metabolism between males and females, which may affect BMI. Patients with HNSCC may experience changes in BMI due to various factors related to the disease and its treatment. A common observation in such patients is weight loss, which can lead to a reduction in BMI. Weight loss in HNSCC patients can occur for several reasons, including tumor-related factors, metabolic changes, and treatment-related factors. Tumor-related factors, such as a tumor in the head and neck region, can affect a patient’s ability to eat, chew, and swallow, leading to reduced food intake and malnutrition. This can cause weight loss and a decrease in BMI. Metabolic changes caused by cancer can alter the body’s metabolism, leading to increased energy expenditure and changes in nutrient utilization, which may contribute to weight loss and a decrease in BMI. Treatment-related factors, such as surgery, radiation therapy, and chemotherapy, can also affect a patient’s nutritional status. For example, radiation therapy to the head and neck area can cause mucositis, dysphagia, and taste changes, making it difficult for patients to eat and maintain their weight. Similarly, chemotherapy can cause nausea, vomiting, and appetite suppression, leading to weight loss. However, further investigation is required to validate these findings and examine the potential of leptin as a biomarker for cachexia in individuals with HNSCC.

## 5. Conclusions

Managing advanced HNSCC remains a challenging issue, and there is no consensus as to the best approach. However, the present study suggests that induction chemotherapy followed by concurrent chemoradiotherapy may improve treatment outcomes in head and neck cancer patients. The treatment protocol appears to be well tolerated by patients, with a manageable toxicity profile and potential improvements in quality of life. Cytokine level changes may be able to serve as biomarkers for treatment response and survival outcomes, and leptin levels may be a potential biomarker for cachexia development in patients with head and neck cancer.

## Figures and Tables

**Table 1:** Clinical characteristics of the patients.

			Number	%
**Gender**				
Males			39	75
Females			13	25
**ANOVA Table between males and females in LSCC and Controls**				
**Sum of Squares**	**d.f.**	**Variance**	**F**	***p***
0.6037	3	0.2012	2.9553	0.0362
**Age**				
Mean (Median)			54.2 ± 16.24 (50.4)	
Range			35–62	
**Evaluable for response to induction chemotherapy**			50	
**Evaluable for response to induction chemotherapy + concomitant chemoradiotherapy**			44	
**Evaluable for response at completion of all therapy**			44	
**Evaluable for toxicity**			52	
**Site**				
Oral cavity			4	7.7
Oropharynx			22	42.3
Hypopharynx			16	30.7
Larynx			10	19.3
**Tumor grade**				
Well differentiated			12	23.1
Moderately differentiated			22	42.3
Poorly differentiated			18	34.6
**ECOG performance status**				
0			42	80.8
1			10	19.2
**Clinical stage**				
**Stage III**			4	7.6
T2N1			0	0
T3N0			2	3.8
T3N1			2	3.8
**Stage IV**			48	92.4
T1N2			2	3.8
T2N2			6	11.6
T3N2			6	11.6
T3N3			4	7.7
T4N0			8	15.4
T4N1			4	7.7
T4N2			14	26.9
T4N3			4	7.7
**Percentage weight loss**				
None			30	57.7
0–5			10	19.2
5–10			4	7.7
>10			8	15.4

**Table 2:** Clinical response.

**Clinical Response after Three Cycles of Induction Chemotherapy (50 Evaluable Patients)**		
	No.	%
Complete response (CR)	8	16
Partial response (PR)	18	36
Stable disease (SD)	18	36
Progressive disease (PD)	6	12
**Clinical response after three cycles of induction chemotherapy and concomitant chemoradiotherapy (44 evaluable patients)**		
CR	26	59.1
PR > 70%	7	15.9
SD	6	13.6
PD	5	11.4
**Clinical response after three cycles of induction chemotherapy, concomitant chemoradiotherapy, and surgery**		
Stopped treatment at protocol completion because of CR	26	59.1
Refused conservative surgery (PR)	2/7	4.5
Not amenable to radical surgery (SD)	2/6	4.5
Died after concomitant chemoradiotherapy (SD)	2	4.5
PD shifted to other treatment protocols	3	6.8

**Table 3:** Toxicity experienced by patients during the chemotherapy induction phase.

Toxicity	Highest National Cancer Institute (NCI) Toxicity Grade									
	Grade 0		Grade I		Grade II		Grade III		Grade IV	
	No.	%	No.	%	No.	%	No.	%	No.	%
**Hemoglobin**	12	23.1	20	38.5	10	19.2	10	19.2	0	0.0
**Granulocytes**	6	11.5	18	34.7	10	19.2	8	15.4	10	19.2
**Platelets**	16	30.8	2	3.8	14	26.8	8	15.4	10	19.2
**Creatinine**	40	76.9	4	7.7	4	7.7	4	7.7	0	0.0
**Nausea/vomiting**	40	76.9	2	3.8	6	11.6	4	7.7	0	0.0
**Diarrhea**	36	69.3	2	3.8	2	3.8	8	15.4	4	7.7
**Mucositis**	14	26.8	8	15.4	8	15.4	16	30.8	6	11.5
**Neurotoxicity**	52	100	0	0.0	0	0.0	0	0.0	0	0.0

**Table 4:** Toxicity experienced by patients during chemoradiotherapy.

Toxicity	Highest NCI Toxicity Grade									
	Grade 0		Grade I		Grade II		Grade III		Grade IV	
	No.	%	No.	%	No.	%	No.	%	No.	%
**Hemoglobin**	2	8.3	8	33.3	4	16.7	4	16.7	6	25
**Granulocytes**	2	8.3	4	16.7	6	25	6	25	6	25
**Platelets**	0	0.0	8	33.3	6	25	4	16.7	6	25
**Creatinine**	24	100	0	0.0	0	0.0	0	0.0	0	0.0
**Nausea/vomiting**	18	75	2	8.3	2	8.3	2	8.3	0	0.0
**Diarrhea**	24	100	0	0.0	0	0.0	0	0.0	0	0.0
**Mucositis**	3	12.5	3	12.5	2	8.4	8	33.3	8	33.3
**Neurotoxicity**	24	100	0	0.0	0	0.0	0	0.0	0	0.0

**Table 5:** Serum levels of cytokines and leptin in healthy controls and HNSCC patients.

Cytokines	Controls (*n* = 50)	Patients before Treatment (*n* = 52)	Student’s *t*-Test	df	*p*-Value
**IL-1β (** **pg/mL)**	3.6 ± 1.2	12.4 ± 4.3	8.9829	98	<0.0001
**IL-2 (** **pg/mL)**	2.31 ± 1.1	14.9 ± 4.1	13.4930	98	<0.0001
**IL-6 (** **pg/mL)**	2.8 ± 1.5	37 ± 14.9	10.2008	98	<0.0001
**TNF α (** **pg/mL)**	2.1 ± 1.7	28 ± 12.9	8.9109	98	<0.0001
**Leptin (** **pg/mL)**	3.1 ± 1.6	19 ± 16.1	3.8366	98	<0.0001

**Table 6:** Cytokine levels (IL-1β, IL-2, IL-6, TNF-α) and leptin in HNSCC patients before and after treatment based on their response to treatment.

Parameters (Mean ± SD)	Complete or Partial Response (*n* = 33)			
	Before Treatment Mean ± SD	After Treatment Mean ± SD	*t*-Test	*p*
**IL-1β** **(pg/mL)**	44.3 ± 31	17.3 ± 3.4	3.8719	0.0004
**IL-2 (** **pg/mL** **)**	29.3 ± 11.7	61.7 ± 37.7	3.671	0.0007
**IL-6 (** **pg/mL** **)**	32 ± 9	14.8 ± 5.7	3.23	0.018
**TNF-α (** **pg/mL** **)**	43.7 ± 14.7	23.2 ± 8.5	5.399	<0.0001
**Leptin (** **pg/mL** **)**	10.7 ± 6.5	16 ± 8.6	2.1987	0.0341
**Parameters** **(mean ± SD)**	**Stable disease (*n* = 6)**			
	**Before treatment** **Mean** **± SD**	**After treatment** **Mean** **± SD**	***t*** **-test**	***p***
**IL-1β** **(pg/mL)**	42.5 ± 2.1	25 ± 4.2	13.94	<0.0001
**IL-2 (** **pg/mL** **)**	53.5 ± 40.3	41.5 ± 4.9	0.5912	0.57
**IL-6 (** **pg/mL** **)**	51.5 ± 14.4	32 ± 8.5	2.857	0.017
**TNF-α (** **pg/mL** **)**	47.5 ± 10.6	35.5 ± 7.8	1.824	0.118
**Leptin (** **pg/mL** **)**	2.3 ± 2	5.6 ± 2.2	2.64	0.014
**Parameters** **(mean ± SD)**	**Progressive disease (*n* = 5)**			
	**Before treatment** **Mean** **± SD**	**After treatment** **Mean** **± SD**	***t*** **-test**	***p***
**IL-1β** **(pg/mL)**	28.7 ± 16.2	34.3 ± 9.6	3.293	0.0081
**IL-2 (** **pg/mL** **)**	31.3 ± 19.1	23.7 ± 5.5	0.937	0.3710
**IL-6 (** **pg/mL** **)**	19 ± 3.6	22.5 ± 4.6	1.467	0.173
**TNF-α (** **pg/mL)**	28.3 ± 11.2	34.7 ± 9.1	1.086	0.303
**Leptin (** **pg/mL** **)**	6.5 ± 4.2	6.1 ± 4.5	0.199	0.85

## Data Availability

The raw data supporting the conclusions of this article will be made available by the authors without undue reservation.
